# Concurrent prenatal diagnosis of hereditary neuropathy with liability to pressure palsies and congenital pulmonary airway malformation: A case report

**DOI:** 10.1097/MD.0000000000051479

**Published:** 2026-11-06

**Authors:** Kai Zhang, JiaTing Yu, Ying He, Mingzhi Ou, Chao Li

**Affiliations:** a Department of General Surgery, Changsha Hospital for Maternal and Child Health Care, Changsha, Hunan, China; b Department of Obstetrics and Gynecology, Changsha Hospital for Maternal and Child Health Care, Changsha, Hunan, China; c Department of Respiratory Medicine, Hunan Provincial People’s Hospital (The First-Affiliated Hospital of Hunan Normal University), Changsha, Hunan, China.

**Keywords:** chromosomal microarray analysis, congenital pulmonary airway malformation, hereditary neuropathy with liability to pressure palsies, *PMP22* gene, prenatal diagnosis

## Abstract

**Rationale:**

Hereditary neuropathy with liability to pressure palsies (HNPP) is a rare autosomal dominant peripheral neuropathy typically diagnosed in adolescence or adulthood. Prenatal diagnosis is exceedingly rare. Congenital pulmonary airway malformation (CPAM) is a common congenital lung lesion. We report a unique case of concurrent prenatal diagnoses of HNPP and CPAM, highlighting the importance of genetic evaluation when structural anomalies are detected prenatally.

**Patient concerns:**

A 38-year-old pregnant woman (gravida 3, para 1) underwent a systematic prenatal ultrasound at 24 weeks of gestation, which revealed a hyperechoic lesion measuring approximately 49 mm × 27 mm × 39 mm in the fetal right thoracic cavity with blood supply from the right pulmonary artery. The pregnancy was otherwise uncomplicated. The fetus’s father and paternal aunt had recurrent limb numbness and weakness, with a known genetic diagnosis of HNPP in the aunt.

**Diagnoses:**

Amniocentesis and chromosomal microarray analysis identified a 1.38 Mb heterozygous deletion at 17p12 encompassing the peripheral myelin protein 22 gene in the fetus, confirming the diagnosis of HNPP. The right-sided thoracic lesion was diagnosed as CPAM based on characteristic ultrasound features.

**Interventions:**

Following multidisciplinary counseling, the parents opted to continue the pregnancy. Individualized perinatal planning was implemented, including elective cesarean delivery at 38 weeks to avoid instrumental extraction and minimize intrapartum nerve compression. Serial ultrasound follow-up was performed every 2 to 3 weeks, and a long-term neurological follow-up plan was established for the newborn.

**Outcomes:**

The patient delivered a healthy female neonate (3150 g, Apgar 9/10) via elective cesarean at 38 + 2 weeks. No neurological or respiratory deficits were observed. CPAM regressed gradually on follow‑up ultrasound. The infant is under regular pediatric and neurological surveillance, with chest imaging scheduled at 6 to 12 months.

**Lessons:**

This case underscores the need for detailed family history and targeted genetic testing when prenatal ultrasound detects structural anomalies. For fetuses with HNPP, individualized perinatal planning – including elective cesarean and long‑term neurological follow‑up – is feasible and recommended. The co‑occurrence of HNPP and CPAM appears coincidental, with no causal link established.

## 1. Introduction

Hereditary neuropathy with liability to pressure palsies (HNPP) is a rare autosomal dominant peripheral neuropathy characterized clinically by recurrent, focal, painless motor and sensory dysfunction in peripheral nerves following minor compression, traction, or trauma.^[1]^ The disease is caused by a heterozygous deletion in the chromosome 17p11.2-p12 region, which contains the peripheral myelin protein 22 gene (*PMP22*), encoding a critical structural component of the peripheral nervous system myelin. Deletion of *PMP22* leads to myelin instability, rendering nerve trunks abnormally sensitive to mechanical stress.^[2]^ Approximately 80% of HNPP cases result from a 1.5 Mb deletion in this region, while the remainder are associated with point mutations in *PMP22*.^[3]^ Notably, duplication of this gene causes a more common hereditary neuropathy, Charcot-Marie-Tooth disease type 1A, forming a disease spectrum based on opposing gene dosage effects.^[4]^

The prevalence of HNPP is estimated at 7 to 16 per 100,000 individuals^[5]^; however, its actual prevalence may be underestimated due to diverse clinical presentations, transient symptoms, and frequent misdiagnosis as other acquired neuropathies. Nerve biopsy, once commonly used for diagnosis, has largely been superseded by genetic testing.^[6]^ HNPP typically manifests in adolescence or early adulthood with recurrent, focal nerve palsies, commonly involving the peroneal, ulnar, median, and brachial plexus nerves.^[7]^ Symptoms usually resolve completely within days to months, and the overall prognosis is favorable, though some patients may experience residual mild dysfunction. As the disease is often diagnosed clinically after symptom onset in adulthood, cases of HNPP diagnosed prenatally are exceptionally rare in the literature.

This report describes a unique fetal case where a right-sided congenital pulmonary airway malformation (CPAM) was identified on prenatal ultrasound. Subsequent amniocentesis and chromosomal microarray analysis (CMA), prompted by a relevant family history, confirmed a diagnosis of HNPP due to a 1.38 Mb heterozygous deletion in the 17p12 region (including *PMP22*). The significance of this case lies in 2 aspects: first, the rarity of prenatally diagnosed HNPP offers a unique perspective on the natural history and early genetic counseling of the condition; and second, the concurrent presence of a neuropathic disorder and a respiratory malformation in the fetus raises questions about whether this represents a coincidental occurrence or a potential genetic link, warranting further exploration. This case is presented in detail (see Table 1).

**Table 1:** Timeline of prenatal diagnosis, genetic confirmation, and perinatal management in a fetus with HNPP and CPAM.

Gestational age	Event	Key details
6–12 wk	First-trimester management	Threatened miscarriage; treated with intramuscular progesterone. Normal NT scan later.
24 wk	Routine anomaly scan	Ultrasound revealed a 49 mm × 27 mm × 39 mm hyperechoic lesion in the right fetal thoracic cavity, suggestive of CPAM.
24 wk (same day)	Family history inquiry	Father and paternal aunt had recurrent limb numbness/weakness and hoarseness; aunt genetically diagnosed with HNPP.
25 wk	Multidisciplinary consultation and amniocentesis	Fetal medicine, genetics, and cardiothoracic surgery teams involved. After counseling, amniocentesis was performed for karyotyping and CMA.
~26 wk	CMA result	1.38 Mb heterozygous deletion at 17p12 encompassing the *PMP22* gene → diagnosis of HNPP confirmed.
26, 29, 32, 35 wk	Serial ultrasound follow-ups	CPAM lesion showed gradual regression. No fetal hydrops, mediastinal shift, or other adverse signs. Normal fetal growth.
35 wk onward	Perinatal planning	Decision to continue pregnancy; elective cesarean section considered to avoid intrapartum nerve compression; absolute contraindication for instrumental delivery; long-term postnatal neurological follow-up planned.

CMA = chromosomal microarray analysis, CPAM = congenital pulmonary airway malformation, HNPP = hereditary neuropathy with liability to pressure palsies, NT = nuchal translucency.

## 2. Case report

A 38-year-old pregnant woman, gravida 3, para 1, with a history of 1 prior induced abortion and no other adverse pregnancy or birth history, was referred. The pregnancy was naturally conceived, with no history of viral infection or radiation exposure during the first trimester. She received intramuscular progesterone for threatened miscarriage from 6 to 12 weeks of gestation, with no other significant medication use. First-trimester nuchal translucency screening was normal; second-trimester maternal serum screening and noninvasive prenatal testing both indicated low risk for aneuploidies. The pregnancy was otherwise uncomplicated, with normal fetal movements.

A systematic prenatal ultrasound at 24 weeks of gestation revealed an abnormal echogenic area within the fetal right thoracic cavity. Ultrasound findings were as follows: a hyperechoic lesion measuring approximately 49 mm × 27 mm × 39 mm with clear borders and homogeneous internal echogenicity was visualized in the right hemithorax. Color Doppler ultrasonography showed blood supply originating from the right pulmonary artery and drainage via the pulmonary vein, consistent with the sonographic features of CPAM (Fig. 1).

**Figure 1.**
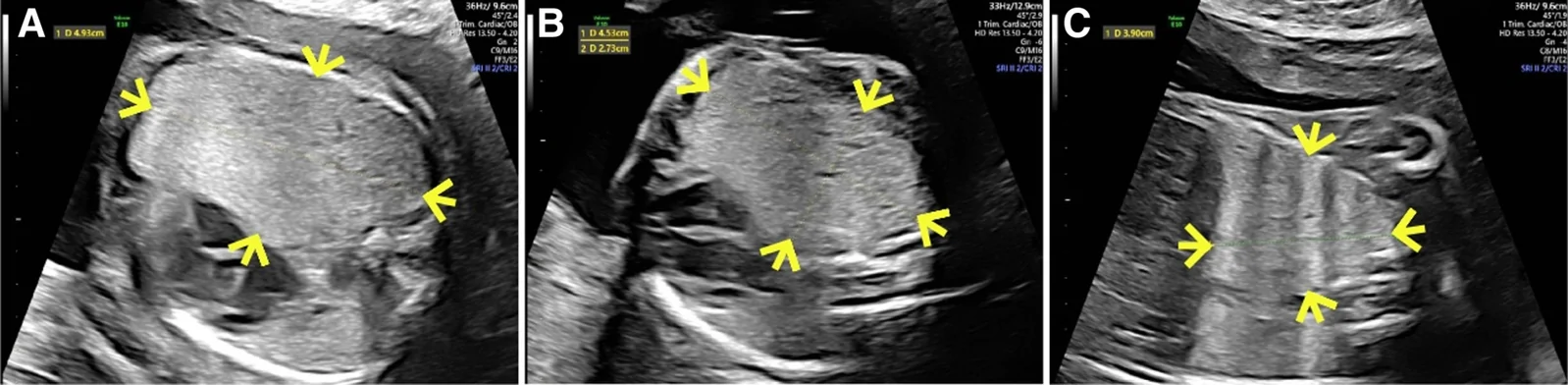
Serial prenatal ultrasound images of the right-sided CPAM lesion. (A–C) Follow-up ultrasound examinations showing the hyperechoic lesion in the fetal right thorax (yellow arrows). Serial measurements demonstrate a gradual reduction in lesion size from (A) approximately 4.93 cm to (B) 4.51 cm and (C) 3.90 cm, consistent with spontaneous regression of the CPAM. CPAM = congenital pulmonary airway malformation.

Due to the fetal pulmonary abnormality, the woman was referred to a fetal cardiopulmonary disorder consultation clinic. Detailed family history inquiry revealed that the fetus’s father and paternal aunt had experienced recurrent episodes of limb numbness and weakness, typically occurring upon waking, lasting 2 to 3 months, and resolving spontaneously. They also experienced recurrent hoarseness. The paternal aunt had been genetically diagnosed with HNPP. This family history raised the possibility of an inherited neurological disorder in the fetus.

Following a multidisciplinary consultation involving prenatal ultrasound experts, fetal medicine specialists, genetic counselors, and cardiothoracic surgeons, a repeat obstetric ultrasound confirmed the right-sided CPAM and revealed no other structural abnormalities. After receiving thorough genetic counseling, the woman underwent amniocentesis for fetal karyotyping and CMA. CMA results showed a heterozygous deletion of approximately 1.38 Mb at 17p12 in the fetus. This region encompasses the *PMP22* gene, and its deletion is a known pathogenic variant causing HNPP. This genetic finding was consistent with the father’s clinical presentation and the aunt’s genetic diagnosis (Fig. 2).

**Figure 2.**
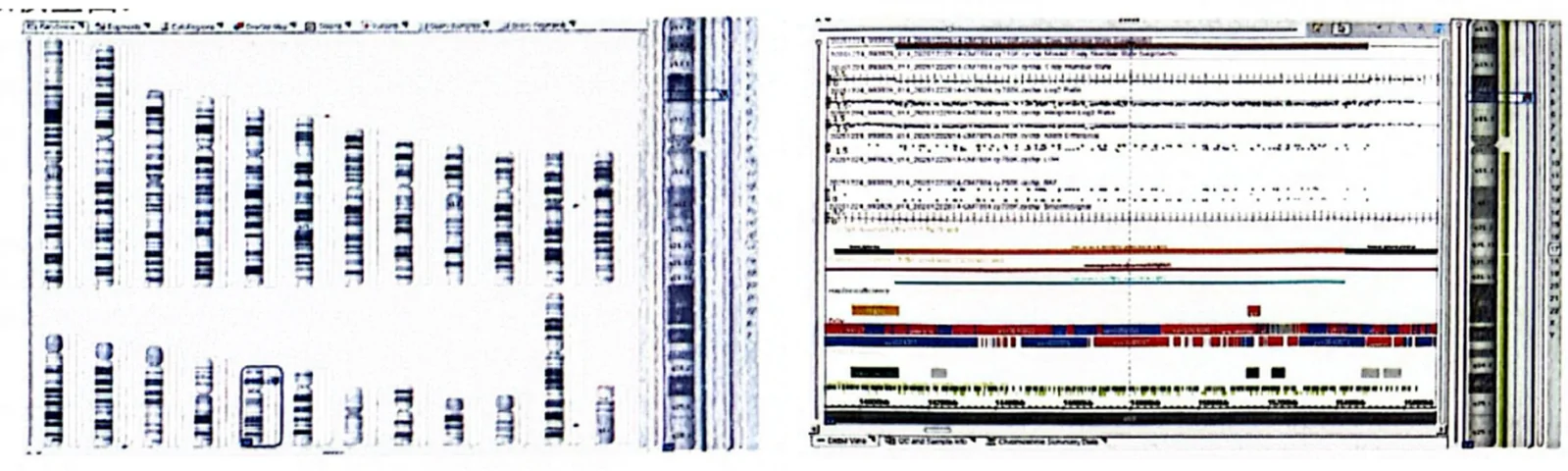
CMA report of the fetus: a 1.38 Mb heterozygous deletion in the 17p12 region (deleted region indicated in red), involving a heterozygous deletion of the region containing the *PMP22* gene. CMA = chromosomal microarray analysis.

Based on the ultrasound and genetic findings, the fetus was diagnosed with HNPP and a right-sided CPAM. Following the diagnosis and further genetic counseling, the parents opted to continue the pregnancy and underwent regular prenatal ultrasound follow-up every 2 to 3 weeks. Subsequent follow-up scans at 26, 29, 32, and 35 weeks of gestation showed normal fetal growth and development. The right-sided CPAM lesion demonstrated a trend towards gradual regression, with no evidence of significant compression on the heart or mediastinum, nor the development of fetal hydrops or other adverse signs (Figs. 3 and 4).

**Figure 3.**
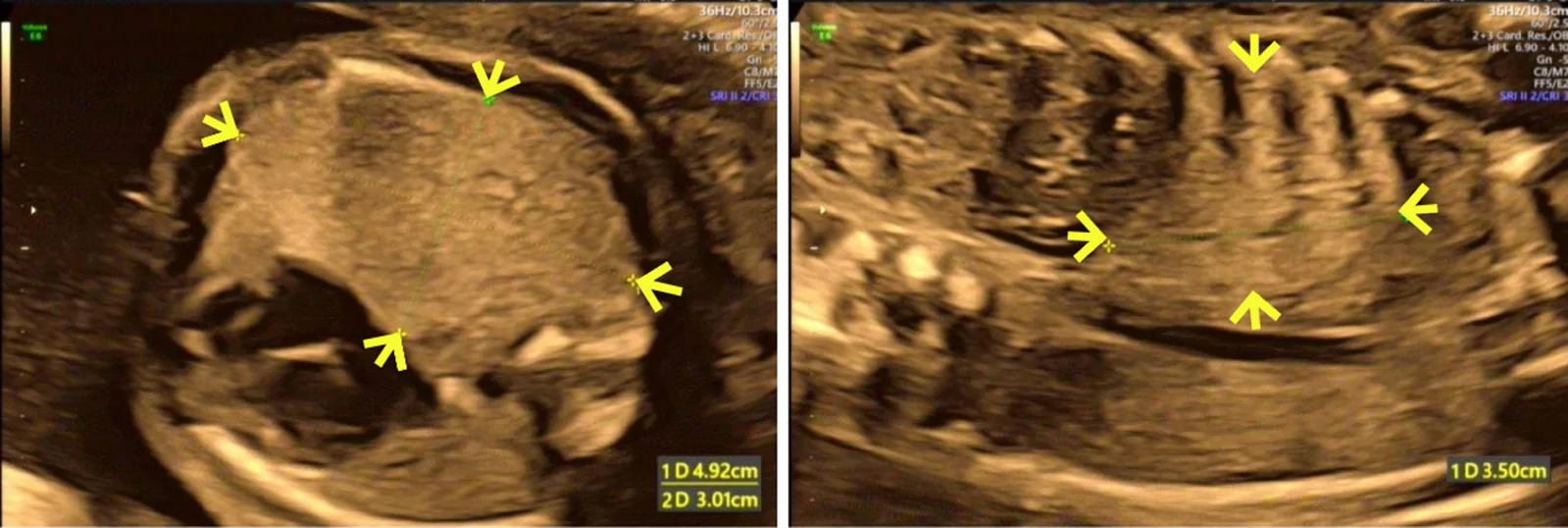
Follow-up ultrasound of the CPAM lesion at 26 weeks of gestation, showing no significant progression in lesion size. CPAM = congenital pulmonary airway malformation.

**Figure 4.**
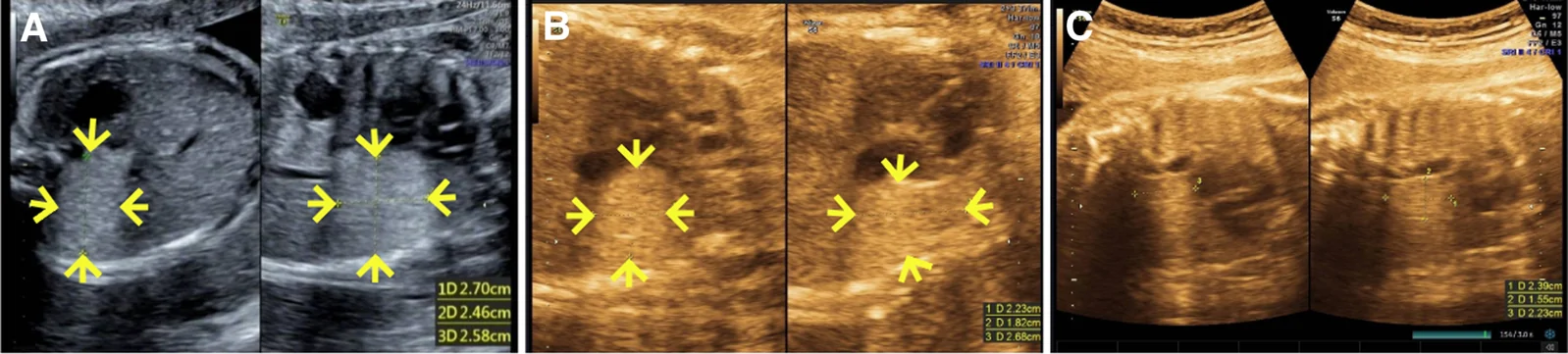
Serial follow-up ultrasound of the CPAM lesion at (A) 29 weeks, (B) 32 weeks, and (C) 35 weeks of gestation, demonstrating a trend of gradual reduction in lesion size. CPAM = congenital pulmonary airway malformation.

The pregnancy was continued to term. At 38 weeks and 2 days of gestation, the patient underwent an elective cesarean section as planned, with the decision based on advanced maternal age and the goal of minimizing intrapartum nerve compression risks, including avoidance of instrumental delivery. The procedure was uneventful, and a healthy female neonate was delivered with a birth weight of 3150 g and Apgar scores of 9 and 10 at 1 and 5 minutes, respectively. Immediate neonatal examination revealed no limb weakness, feeding difficulties, or respiratory distress. A baseline neurological examination was normal. The infant was discharged home in good condition and is currently under regular pediatric and neurological follow-up, with postnatal chest imaging planned at 6 to 12 months of age to confirm the CPAM diagnosis and guide further management.

## 3. Discussion

This report presents a case of fetal HNPP diagnosed prenatally, co-occurring with a right-sided CPAM. To the best of our knowledge, cases of HNPP diagnosed during the fetal period are extremely rare in the literature, and the concurrent occurrence of HNPP and CPAM has not been previously reported. The uniqueness of this case provides valuable insights into the natural history and genetic counseling of HNPP, as well as the potential relationship between these 2 conditions.

Although the co‑occurrence of HNPP and CPAM in this case appears coincidental, we believe this report offers distinct clinical value in 3 aspects: it demonstrates the importance of eliciting a detailed family history when prenatal ultrasound identifies structural anomalies; it illustrates the utility of CMA in the prenatal setting for diagnosing unexpected genetic conditions that may otherwise remain undetected until symptomatic presentation in adolescence or adulthood; and it provides a practical framework for perinatal management of fetuses with HNPP, including delivery planning and neonatal care strategies. These practical takeaways are directly applicable to prenatal counseling and obstetric decision‑making.

HNPP is an autosomal dominant disorder caused by a heterozygous deletion in the chromosome 17p11.2-p12 region, which includes the *PMP22* gene. *PMP22* is a critical component of peripheral nervous system myelin; its deletion results in myelin instability and increased susceptibility of nerve trunks to mechanical stress.^[2]^ The diagnosis in this fetus was confirmed by CMA, which identified a 1.38 Mb heterozygous deletion at 17p12 encompassing *PMP22*, consistent with the typical genetic features of HNPP. The family history in this case is highly characteristic of HNPP: the father and paternal aunt exhibited recurrent episodes of limb numbness and weakness, often upon waking, lasting 2 to 3 months with spontaneous resolution, along with recurrent hoarseness. This pattern of transient, recurrent, compression-induced neurological symptoms is a hallmark of HNPP.^[1]^ Notably, the clinical presentation of HNPP is heterogeneous; some individuals may remain asymptomatic for long periods, and the condition is often misdiagnosed as other acquired neuropathies, potentially leading to an underestimation of its true prevalence. Although this fetus did not display symptoms in utero, the presence of the pathogenic variant indicates a clear risk for developing compression palsies later in life.

Prenatal diagnosis of HNPP is exceptionally rare, as the condition is typically diagnosed clinically in adolescence or early adulthood upon the emergence of symptoms. The definitive diagnosis in this case holds significant implications for perinatal management and long-term follow-up. Delivery planning should focus on minimizing mechanical compression, traction, and procedure-related stimuli during labor that could precipitate or exacerbate nerve injury in a newborn carrying a *PMP22* deletion. Key considerations include evaluating the likelihood of a smooth labor process. If factors such as prolonged labor or inadequate uterine contractions are anticipated, elective cesarean section should be prioritized. Regardless of delivery mode, prolonged fixed positioning should be avoided, and soft padding should be applied to pressure points. Instrumental deliveries (e.g., forceps, vacuum extraction) are an absolute contraindication in HNPP deliveries due to the high risk of nerve injury.^[8,9]^ Regional anesthesia (spinal/epidural) is preferred, with careful attention to anesthetic technique.^[10]^ If the mother has a history of prior delivery-related nerve palsy or if labor abnormalities are anticipated, an elective cesarean section should be considered directly. Neonatal management is also crucial. The infant may be asymptomatic at birth, but parents must be counseled about the hereditary nature of HNPP, its long-term manifestations, and precautions. Avoiding excessive limb compression during the neonatal period and throughout childhood is essential. While routine genetic testing for asymptomatic HNPP neonates is not currently recommended, establishing a long-term neurological follow-up plan is necessary to monitor for symptom onset and enable early intervention. A baseline neurological examination was performed in the neonatal period and was normal; however, given the typical adolescent‑ or adult‑onset of HNPP symptoms, the infant has been enrolled in a long‑term follow‑up program with annual neurological assessments to monitor for symptom onset and enable early intervention.

The planned elective cesarean delivery was successfully implemented, and the absence of neonatal neurological deficits supports the feasibility of individualized perinatal planning for fetuses diagnosed with HNPP. This experience reinforces the recommendation that delivery planning should prioritize minimizing mechanical compression and avoiding instrumental extraction.

The other unique aspect of this case is the concurrent presence of a right-sided CPAM. CPAM is a rare congenital lung malformation characterized by overgrowth and cystic dilatation of terminal bronchioles, with an estimated incidence of 1 in 10,000 to 35,000 live births.^[11]^ The genetic etiology of CPAM remains incompletely understood; recent whole-exome sequencing studies suggest associations with various genes involved in embryonic development, epithelial barrier function, and immune-metabolic pathways.^[12,13]^

Based on current evidence and the findings in this case, the co-occurrence of HNPP and CPAM is more likely coincidental rather than directly linked genetically. The primary function of *PMP22* is encoding a structural protein in peripheral myelin, and no direct evidence implicates this gene in lung development or airway branching morphogenesis.^[14,15]^ Furthermore, the typical HNPP deletion region (17p12) does not contain known genes essential for lung development. Importantly, the CPAM in this case was an isolated finding that remained stable or regressed during follow‑up, consistent with the natural history of most CPAM lesions,^[16]^ further supporting the likelihood of a coincidental association. While the statistical improbability of 2 rare conditions co‑occurring is acknowledged, this case does not establish a novel syndromic entity. Rather, it serves as a reminder that when multiple anomalies are encountered, comprehensive genetic evaluation may reveal unexpected diagnoses, and not all concurrent findings are pathogenetically linked.

This case report has several limitations. First, although HNPP was confirmed by CMA – currently considered the diagnostic gold standard for this condition – postnatal neurological phenotyping has not yet been performed beyond the neonatal baseline examination, as the infant remains asymptomatic in the neonatal period. Long‑term neurological follow‑up is ongoing and will be essential to correlate the genotype with the eventual clinical phenotype. Second, the CPAM diagnosis was based solely on prenatal ultrasound findings without histopathological confirmation, as there was no clinical indication for surgical resection; postnatal chest imaging is planned at 6 to 12 months of age to provide further diagnostic validation. It is worth noting that prenatal ultrasound features of CPAM – particularly a well‑defined hyperechoic lesion with pulmonary arterial blood supply – have a high positive predictive value when evaluated by experienced fetal ultrasound specialists. Third, whole-exome sequencing was not performed on the fetus, preventing a comprehensive exclusion of other single-gene pathogenic variants associated with CPAM and limiting a more thorough genetic analysis of the relationship between the 2 conditions.

## 4. Conclusion

This case reports the first prenatal diagnosis of HNPP concurrent with CPAM. CMA identified a heterozygous deletion in the *PMP22* gene at 17p12, and the diagnosis was confirmed in conjunction with the family history. This case highlights that when structural abnormalities are detected on prenatal ultrasound, a detailed family history should be obtained and targeted genetic evaluation promptly considered. For fetuses diagnosed with HNPP, individualized perinatal management – including elective cesarean delivery to avoid instrumental extraction and minimize nerve compression, as well as long‑term neurological follow‑up – is both feasible and recommended. The co‑occurrence of HNPP and CPAM in this case appears coincidental, and no causal link is established. For fetuses presenting with multiple anomalies, comprehensive genetic testing such as whole‑exome or whole‑genome sequencing should be considered when available to provide a more robust foundation for etiological analysis and clinical counseling.

## Acknowledgments

The authors express their gratitude to the patient for consenting to the publication of her medical data. The authors also thank the staff of the Department of General Surgery, Obstetrics and Gynecology, and the Ethics Committee of Changsha Hospital for Maternal and Child Health Care for their support.

## Author contributions

**Conceptualization:** Chao Li, Kai Zhang, JiaTing Yu.

**Data curation:** Chao Li, Kai Zhang.

**Project administration:** Chao Li.

**Software:** Kai Zhang, Ying He, Mingzhi Ou.

**Formal analysis:** JiaTing Yu, Ying He, Mingzhi Ou.

**Investigation:** JiaTing Yu.

**Supervision:** Ying He.

**Methodology:** Mingzhi Ou.

**Writing – original draft:** Chao Li, Kai Zhang.

**Writing – review & editing:** Chao Li.
